# Indoleamine 2,3-dioxygenase regulates anti-tumor immunity in lung cancer by metabolic reprogramming of immune cells in the tumor microenvironment

**DOI:** 10.18632/oncotarget.12249

**Published:** 2016-09-26

**Authors:** Cara C. Schafer, Yong Wang, Kenneth P. Hough, Anandi Sawant, Stefan C. Grant, Victor J. Thannickal, Jaroslaw Zmijewski, Selvarangan Ponnazhagan, Jessy S. Deshane

**Affiliations:** ^1^ Department of Medicine, University of Alabama at Birmingham, Birmingham, AL, USA; ^2^ Department of Pathology, University of Alabama at Birmingham, Birmingham, AL, USA

**Keywords:** indoleamine 2, 3-dioxygenase, myeloid-derived suppressor cells, lung cancer, metabolism, combination therapy

## Abstract

Indoleamine 2,3-dioxygenase (IDO) has been implicated in immune evasion by tumors. Upregulation of this tryptophan (Trp)-catabolizing enzyme, in tumor cells and myeloid-derived suppressor cells (MDSCs) within the tumor microenvironment (TME), leads to Trp depletion that impairs cytotoxic T cell responses and survival; however, exact mechanisms remain incompletely understood. We previously reported that a combination therapy of gemcitabine and a superoxide dismutase mimetic promotes anti-tumor immunity in a mouse model of lung cancer by inhibiting MDSCs, enhancing polyfunctional response of CD8^+^ memory T cells, and extending survival. Here, we show that combination therapy targets IDO signaling, specifically in MDSCs, tumor cells, and CD8^+^ T cells infiltrating the TME. Deficiency of IDO caused significant reduction in tumor burden, tumor-infiltrating MDSCs, GM-CSF, MDSC survival and infiltration of programmed death receptor-1 (PD-1)-expressing CD8^+^ T cells compared to controls. IDO^−/−^ MDSCs downregulated nutrient-sensing AMP-activated protein kinase (AMPK) activity, but IDO^−/−^ CD8^+^ T cells showed AMPK activation associated with enhanced effector function. Our studies provide proof-of-concept for the efficacy of this combination therapy in inhibiting IDO and T cell exhaustion in a syngeneic model of lung cancer and provide mechanistic insights for IDO-dependent metabolic reprogramming of MDSCs that reduces T cell exhaustion and regulates anti-tumor immunity.

## INTRODUCTION

The metabolic state and activities of the immune cells comprising the tumor microenvironment (TME) influences the proliferative and invasive capacity of tumor cells. Tumors evade immune detection through various mechanisms of immune suppression including upregulation of indoleamine 2,3-dioxygenase (IDO), a heme-containing enzyme that catalyzes the conversion of the essential amino acid tryptophan (Trp) into the metabolic byproduct kynurenine (Kyn) [1]. IDO overexpression, in both hematopoietic and non-hematopoietic compartments [2], can alter metabolic properties of immune and tumor cells to promote tumor progression [3]. Increased IDO expression and activity are correlated with poor patient prognosis and quality of life [4, 5]. IDO has been identified as a pathogenic driver of MDSC expansion leading to cancer progression by mediating IL-6 driven MDSC suppressive function [6]. Not only does IDO activation deplete essential Trp from the TME, but it also causes accumulation of Kyn which generates a tumor-promoting environment by converting naïve CD4^+^ T cell development into regulatory T cells (T_regs_) that facilitate tolerance to tumors and counteract anti-tumor immune cells [1, 7]. In addition to IDO, upregulation of programmed death ligand-1 and its engagement with the inhibitory receptor programmed death receptor-1 (PD-1) have also been identified as immune inhibitory mechanisms that downregulate active T cell responses [8, 9].

Recent studies investigating nutrient-signaling responses to Trp availability have identified independent roles for mechanistic target of rapamycin (mTOR) and general control non-derepressible 2 (GCN2) kinases in cancer cells [10, 11]. When Trp levels are sufficient (Trp sufficiency), activated mTOR phosphorylates ribosomal S6 kinase, which phosphorylates downstream ribosomal protein S6 (pS6) [12]. S6 activation turns on cellular growth, proliferation, and protein synthesis [13]. When Trp levels are reduced following induction of IDO [3], resulting in Trp deficiency, stores of uncharged Trp-tRNA become elevated and this prompts the integrated stress response to stimulate GCN2 kinase [14]. Activated GCN2 subsequently phosphorylates eukaryotic initiation factor 2 alpha (peIF2α), limiting cell growth and protein translation [14].

Alternate mechanisms involving nutrient sensor AMP-activated protein kinase (AMPK) can control mTOR, most notably, by activating mTOR inhibitory complexes [18, 19]. In cancer, AMPK serves as a key metabolic regulator in maintaining energy homeostasis during cellular stress [18]. AMPK functions to restore depleted ATP levels [20] and becomes activated when upstream liver kinase B1 (LKB1) tumor suppressor is phosphorylated [18]. Activated AMPK has different downstream functions, including phosphorylation of 6-phosphofructo-2-kinase/fructose-2,6-biphosphatase 2 (pPFKFB2), an enzyme involved in glycolytic flux [21]. Since AMP-activated protein kinase (AMPK) and mTOR are key regulators of nutrient-sensing [18], Trp availability will influence these pathways. AMPK has been shown to have both pro-tumorigenic and anti-tumorigenic roles in cancer [22], but mechanisms by which AMPK influences tumor cell responses remain unclear. In CD8^+^ T cells, AMPK has been shown to be essential for survival and memory recall [23].

We previously demonstrated the effectiveness of a combination therapy of gemcitabine (GEM) and a superoxide dismutase mimetic (SOD) in reducing tumor infiltrating MDSCs and T_regs_, while enhancing the quantity and quality of cytotoxic CD8^+^ T cell response in the TME, in a murine model of lung cancer [15]. Since IDO is expressed in immunosuppressive cell types, including MDSCs [9, 16] and T_regs_ [17] as well as tumors, we explored the effects of inhibition of IDO pathway in these cells.

In this study, we demonstrated that our combination therapy significantly inhibited IDO, mTOR, and AMPK pathways in mice injected with Lewis lung carcinoma cells (LLCs). We investigated the impact of IDO on these signaling pathways in MDSCs, LLCs, and CD8^+^ T cells purified from the TME of wild type (WT) and IDO-deficient (IDO^−/−^) mice. We identified that IDO deficiency not only diminished tumor burden, but also significantly limited the infiltration of MDSCs in tumor tissue. Reduced infiltration of both the PD-1^hi^ CD4^+^ and CD8^+^ T cells was noted in IDO^−/−^ mice. AMPK activation was reduced in IDO-deficient MDSCs compared to WT MDSCs. In contrast, tumor-infiltrating CD8^+^ T cells in IDO-deficient mice showed activation of AMPK with enhanced IFN-γ and lactate production. We report that combination therapy reduced IDO and mTOR pathway activation in MDSCs and LLCs and further significantly enhanced IFN-γ and glycolytic lactate production by CD8^+^ T cells. These data suggest that direct targeting of MDSCs and lung cancer cells by combination therapy can inhibit IDO signaling and tumor cell proliferation pathways, while reducing T cell exhaustion and promoting enhanced glycolytic metabolism of cytotoxic CD8^+^ T cells.

Taken together, this study reveals a novel role for IDO in regulation of AMPK activation, distinct from Trp sufficiency and deficiency signaling through mTOR and GCN2. These data also provide mechanistic evidence that a combination treatment of gemcitabine and a SOD mimetic can metabolically reprogram the cellular components of the TME including suppressor cells, effector T cells, and tumor cells.

## RESULTS

### Combination therapy inhibits IDO pathway and inhibition of IDO impairs tumor growth and tumor infiltration of MDSCs

**Figure 1:**
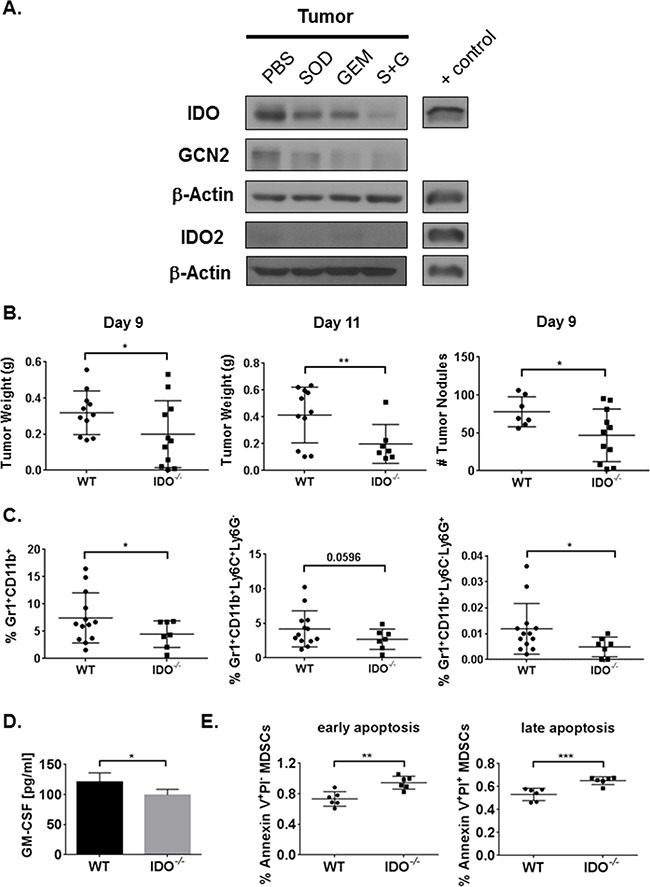
Combination therapy reduces tumor IDO expression and IDO deficiency inhibits tumor burden along with MDSC infiltration and survival in the TME Mice were injected *i.c.* with 1×10^6^ LLCs and treated with PBS, SOD mimetic (SOD), gemcitabine (GEM), or SOD mimetic and gemcitabine (S+G). Tumor lysates were collected on day-9 for Western Blot analysis. **A.** IDO pathway is inhibited in tumor by combination treatment. **B.** WT mice have larger tumors and more nodules compared to IDO^−/−^ mice (three pooled independent experiments, n=7-11 mice/group) on day-9 and day-11 post-*i.c.* injection analyzed by student's unpaired t-test. **C.** By flow cytometry, total percentages of tumor-infiltrating MDSCs from the live cell gate, and both monocytic and granulocytic MDSCs, are diminished in IDO^−/−^ mice (pooled independent experiments, n=7-13 mice/group) on day-11 post-*i.c.* injection. Data in B and C are compared using a student's unpaired t-test with Welch's correction, *P<0.05, **P<0.001. In lung homogenates from day-11 post-tumor implant, **D.** IDO^−/−^ mice (n=4) exhibit lower ELISA concentrations of GM-CSF compared to WT (n=3). **E.** By flow cytometry, IDO-deficient bone marrow-differentiated MDSCs demonstrate higher total percentages of apoptotic MDSCs (6 replicates/group). Data in D and E are analyzed by student's unpaired t-test, *P<0.05, **P<0.005, ***P<0.001.

We previously reported that infiltration of Gr1^+^CD11b^+^ MDSCs increased with tumor progression in a syngeneic, intracardiac (*i.c.*) murine model of lung cancer [15]. Specifically, combination therapy (S+G) of gemcitabine (GEM) and a SOD mimetic (SOD) inhibited tumor-infiltrating MDSCs [15]. This enhanced anti-tumor immunity by promoting proliferation and persistence of multi-functional CD8^+^ T cells, resulting in prolonged survival [15]. To investigate the mechanisms underlying this enhanced cytotoxic CD8^+^ T cell response, we first determined if combination therapy inhibits IDO pathway in the TME. After transplant of LLCs into WT mice, tumors grew rapidly over two weeks (Supplementary Figure S1A). Tumor burden was reduced following administration of combination therapy (Supplementary Figure S1B). Immunoblot analyses showed reduction of IDO expression in tumor (Figure 1A, Supplementary Figure S1C), lung, and spleen tissues (Supplementary Figure S1D). In the tumor tissue, expression of IDO1 was greater than its isoform IDO2 (Figure 1A), suggesting a regulatory role for IDO1 in mediating immunosuppression and inducing downstream GCN2 expression. Additionally, serum IDO activity was reduced following combination therapy (Supplementary Figure S1E) compared to gemcitabine-treated controls. The significant reduction of IDO activity and lung IDO expression by the SOD mimetic alone may also reflect a unique advantage of this antioxidant mimetic in altering IDO function in oxidant-rich lung tumors.

Tumor-promoting MDSCs and tumor cells expressing IDO can enhance tumor growth [24–27]. We evaluated IDO expression in MDSCs and tumor cells from the TME. Immunoblot analyses showed predominant IDO expression in the tumor nodules and in the purified tumor-associated Gr1^+^CD11b^+^ MDSCs from WT mice (Supplementary Figure S2A), while IDO expression was reduced in the Gr1^−^CD11b^−^ population, representing all other cells in the TME including transplanted tumor cells. To determine the impact of IDO on tumor growth, we confirmed that IDO1, not IDO2, was induced following tumor establishment in the lungs of both WT and IDO-deficient mice (Supplementary Figure S2B). Since all host tissues and tumor-infiltrating immune cells lack *Ido1* in IDO^−/−^ mice, these data suggest that only the transplanted LLC tumor cells contribute to IDO expression in the IDO^−/−^ mice. IFN-γ, a known stimulator of IDO, activates the JAK/STAT pathway to regulate IDO at both the transcriptional and translational level [28]. Although baseline IDO expression was undetectable in LLCs, IDO was induced in LLCs treated with recombinant mouse IFN-γ (Supplementary Figure S2C), suggesting that cytokines and other factors in the TME can induce IDO in tumor cells transplanted into IDO-deficient mice. There was no difference in IFN-γ production comparing tumor-bearing WT and IDO^−/−^ mice (Supplementary Figure S2D). As tryptophan dioxygenase (TDO) is another enzyme that may produce kynurenine, we investigated TDO2 expression in the lungs of tumor bearing WT and IDO^−/−^ mice. As shown in Supplementary Figure S2E, although TDO2 expression was noted in the naïve lung tissues of WT and IDO^−/−^ mice, significantly reduced expression was observed in tumor bearing mice.

At day-9, IDO-deficient mice exhibited diminished tumor burden and fewer tumor nodules (Figure 1B). Even at day-11, tumor burden was reduced in mice lacking IDO (Figure 1B). Therefore, IDO expression from transplanted LLCs in the IDO-deficient mice was not sufficient to promote tumor growth, validating the predominant role for IDO-expressing MDSCs in enhancing tumor growth. Similar results were also observed using an intravenous model of tumor implantation (Supplementary Figure S3A). We then investigated whether IDO deficiency would impact immune cell infiltration in the TME. Tumor infiltration of total immunosuppressive MDSCs, and percentages of both granulocytic (Ly6G^+^Ly6C^−^) and monocytic (Ly6G^−^Ly6C^+^) MDSC subsets, were diminished in IDO^−/−^ mice (Figure 1C and Supplementary Figure S3B). Similarly, our combination therapy also reduced the percentages of MDSCs in tumor, lung, and spleen tissues [15]. Levels of granulocyte-macrophage colony-stimulating factor (GM-CSF), a pro-inflammatory cytokine known to drive MDSC differentiation and expansion [29, 30], were reduced in lung tissues from tumor-bearing IDO^−/−^ mice compared to WT controls (Figure 1D). Lower GM-CSF concentrations could account for diminished presence of MDSCs in IDO^−/−^ mice. Since GM-CSF levels were altered, we determined the impact of IDO on overall survival of MDSCs. In *in vitro*-differentiated MDSCs from bone marrow, increase in MDSC cell death, characterized by Annexin V and PI staining was noted in IDO^−/−^ mice (Figure 1E). Together, these data demonstrate a critical role for IDO in MDSC infiltration and survival in LLC tumor-bearing mice.

### IDO deficiency limits CD8^+^ and CD4^+^ T cell exhaustion in tumor-bearing mice

**Figure 2:**
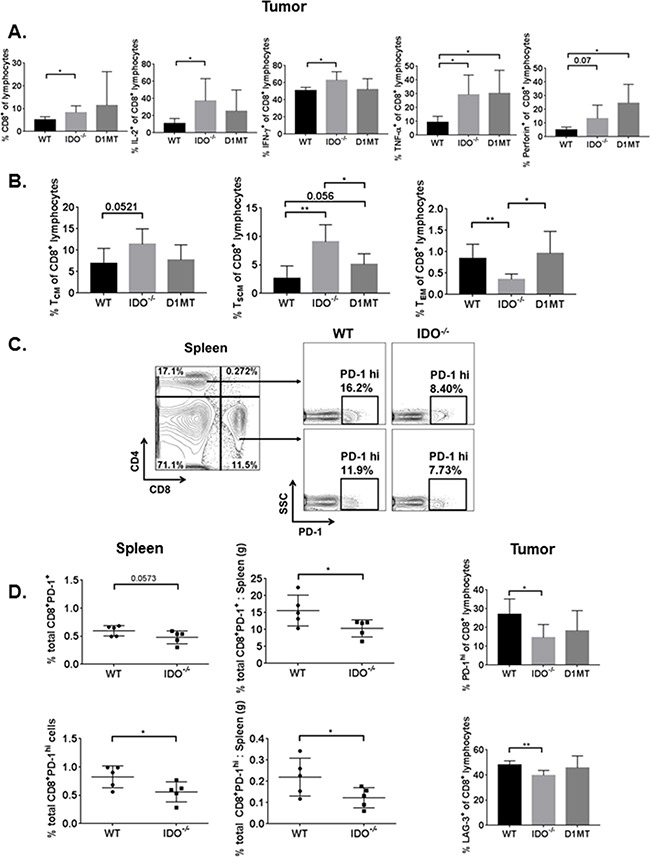
Tumor-bearing IDO^−/−^ T lymphocytes are more polyfunctional, have enhanced memory, and express fewer T cell surface exhaustion markers On day-9 from *i.c.*-injected WT, IDO^−/−^, and D1MT treated (n=4-5 mice/group), tumor cells were analyzed by flow cytometry for intracellular cytokines and memory T cell subsets, as described. The percentages are calculated for **A.** IL-2^+^, IFN-γ^+^, TNF-α^+^, and perforin^+^ CD8^+^ T lymphocytes, with IDO^−/−^ T cells exhibiting greater percentages of these polyfunctional cytokines in the TME. Data are analyzed by student's unpaired t-test, *P<0.05. **B.** Compared to WT, percentages of central memory (T_CM_) and stem cell memory (T_SCM_) CD8^+^ T lymphocytes are elevated in IDO^−/−^ mouse tumors, while effector memory CD8^+^ T lymphocyte (T_EM_) subsets are reduced in IDO^−/−^ mice. For T_CM_, WT vs IDO^−/−^ data are analyzed by student's unpaired t-test. For T_SCM_, WT vs IDO^−/−^ and IDO^−/−^ vs D1MT are compared by student's unpaired t-test and WT vs D1MT is compared by Mann-Whitney, *P<0.05, **P<0.005. For T_EM_, WT vs IDO^−/−^ data are analyzed by Mann-Whitney and WT vs D1MT values are compared by student's unpaired t-test, *P<0.05, **P<0.01. On day-9 following tumor *i.c.* implant in WT and IDO^−/−^ mice (n=5 mice/group), spleens were analyzed for PD-1 surface expression on CD4^+^ and CD8^+^ T cells, as demonstrated by the gating strategy in **C. D.** IDO*^−/^* mice show much lower total PD-1^+^ and PD-1^hi^ percentages for CD8^+^ T cells (and as a ratio to corresponding spleen weight). Similarly, in the tumor, IDO deficiency impairs the percentages of PD-1^hi^ and LAG-3^+^ surface expression on CD8^+^ T cells. Data are analyzed by student's unpaired t-test, *P<0.05, **P<0.01.

To determine the impact of IDO deficiency on immunosuppression by MDSCs, we measured tumor-infiltrating CD8^+^ T cells. Percentages of CD8^+^ T cells and percentages of polyfunctional effector T cells producing effector cytokines IL-2, IFN-γ, and TNF-α were significantly elevated in IDO^−/−^ mice, (Figure 2A). IDO deficiency also elevated tumor-infiltrating central memory and stem cell memory T cells (T_CM_ and T_SCM_) but caused a reduction of effector memory T cells (T_EM_) (Figure 2B). Treatment with IDO inhibitor, 1-methyl-D-tryptophan (D1MT), enhanced percentages of only TNF-α^+^ and perforin^+^ CD8^+^ T cells compared to IDO deficient mice. Similarly, although D1MT treatment enhanced effector memory CD8^+^ T cells compared to IDO^−/−^ mice, it did not have comparable effects for induction of the other memory cell subsets. Because IDO-expressing MDSCs induce suppressive mechanisms to downregulate T cell function and promote anergy [31], we investigated whether CD8^+^ T cell exhaustion was alleviated in IDO-deficient mice. We evaluated expression of checkpoint molecules PD-1, LAG-3, TIM-3, and CTLA-4. Figure 2C and 2D show that PD-1 expression and percentages of the CD4^+^ and CD8^+^PD-1^hi^ T cells (Supplementary Figure S4A), were reduced in the spleens of tumor-bearing IDO-deficient mice, as well as the total percentages and ratio of total percentages to spleen weights of PD-1^+^ and PD-1^hi^ CD8^+^ T cells. These results suggest that checkpoint molecule PD-1 is impaired on CD8^+^ lymphocytes in both the spleen and tumor tissues which may dampen T cell exhaustion to promote effector T cell polyfunctionality in the TME. Additionally, in the tumors, percentages of CD8^+^LAG-3^+^ T cells were also reduced in IDO^−/−^ mice, without any significant differences in additional checkpoint molecules TIM-3 and CTLA-4 (Supplementary Figure S4B). These data emphasize the importance of targeting IDO-expressing MDSCs to overcome T cell exhaustion.

### Tumor-derived MDSCs lacking IDO exhibit reduced activation of AMPK pathway

**Figure 3:**
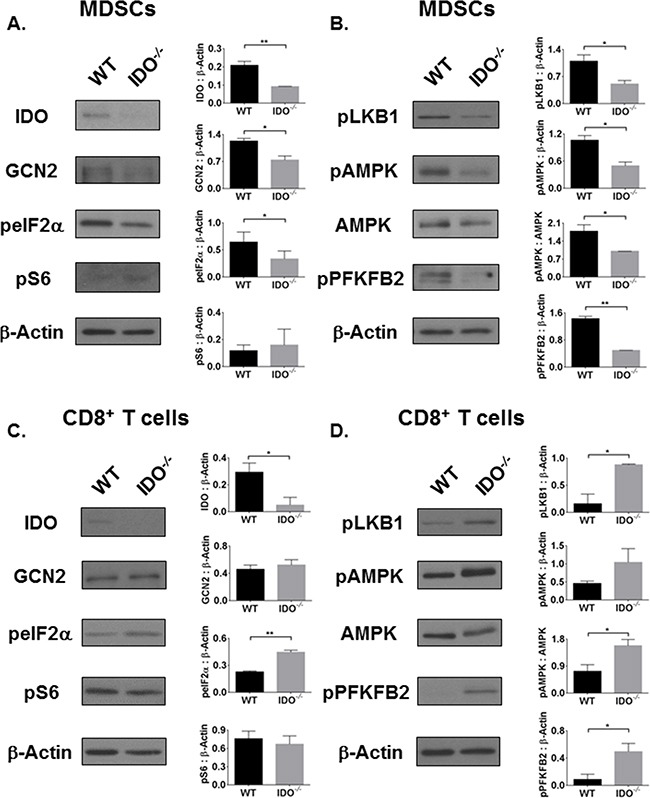
The AMPK pathway is inversely activated in IDO-deficient MDSCs and CD8^+^ T cells, independent of mTOR activation status On day-11, CD45^+^Gr1^+^CD11b^+^ MDSCs were FACS-purified from *i.c.* tumors of WT and IDO^−/−^ mice and analyzed by Western Blot. **A.** IDO and GCN2 as well as **B.** AMPK pathway activation are reduced in IDO-deficient MDSCs, independent of mTOR. In addition, CD45^+^CD8^+^ T cells were FACS-purified from tumors and expanded. **C.** Although GCN2 expression remains unchanged in IDO^−/−^ CD8^+^ T cells, eIF2α is activated. No difference in mTOR activation marker pS6 is observed. **D.** AMPK pathway is activated in IDO^−/−^ CD8^+^ T cells. Densitometry data, as a ratio of (phosphorylated) protein to β-Actin, are from at least two experimental replicates and compared by student's unpaired t-test, *P<0.05, **P<0.01.

Next, we investigated mechanisms of metabolic modulation by IDO. MDSCs and tumor cells in the TME can both alter Trp metabolism by upregulating IDO to induce tolerance to tumor-specific T cells [25, 26, 32]. Over-activation of IDO inhibits the mTOR pathway which regulates Trp sufficiency signaling [10, 33, 34]. We first examined how cellular mTOR phosphorylation of S6 (Trp sufficient signaling) and how cellular GCN2 phosphorylation of eIF2α (Trp deficient signaling) would be affected in the absence of host IDO. By fluorescence-activated cell sorting (FACS), MDSCs were characterized by initially gating on CD45, or leukocyte common antigen, expressed only on cells of hematopoietic origin. From the CD45^+^ population, MDSCs were positively identified by Gr-1^+^CD11b^+^ surface expression while T cells (in the lymphocyte gate) were characterized by surface expression of CD8. We immunosorted CD45^+^Gr1^+^CD11b^+^CD8^−^ MDSCs from the tumor tissues of WT and IDO^−/−^ mice. As shown in Figure 3A, in IDO^−/−^ MDSCs, there was a significant reduction in GCN2 expression which led to decreased activation of Trp deficiency signal, measured by phosphorylation of eIF2α (peIF2α). MDSCs showed no difference in activation of mTOR effector molecule ribosomal S6 (pS6) in both strains of mice, suggesting that Trp sufficiency and deficiency signaling is independently regulated by IDO. MDSCs from IDO^−/−^ mice exhibited reduced activation of the AMPK pathway, including upstream LKB1 and downstream PFKFB2 (Figure 3B). MDSCs isolated from the bone marrow of tumor-bearing IDO^−/−^ mice also showed diminished activation of AMPK compared to WT (Supplementary Figure S5). These data suggest the possibility of an inherent deficit in AMPK activation in IDO-deficient MDSCs that is not limited to AMPK impairment within MDSCs residing in the TME.

### AMPK signaling and effector functions of tumor-specific CD8^+^ T cells are turned on in IDO-deficient mice

Because IDO-expressing MDSCs contribute to CD8^+^ T cell tolerance, we investigated how IDO deficiency influenced metabolic signaling in CD8^+^ T cells within the TME. Here, we FACS-purified CD45^+^CD8^+^Gr-1^−^CD11b^−^ T cells from the tumor tissue. As shown in Figure 3C, the absence of IDO in CD8^+^ T cells did not influence GCN2 expression. In fact, eIF2α activation in these cells was increased without affecting mTOR phosphorylation of S6. There was no difference in expression or activation of IDO, GCN2, or mTOR pathway in CD45^−^ LLCs (Supplementary Figure S6), suggesting that the IDO-deficient immune cells are responsible for altered Trp signaling rather than the tumor cells. Since Trp deficiency signaling through eIF2α was independent of Trp sufficiency signaling via mTOR in MDSCs, CD8^+^ T cells, and LLCs, this indicates an alternate pathway may be regulating metabolic signaling cues within these cells.

Knowing that Trp metabolism can influence downstream signaling we asked how IDO regulates nutrient metabolism in these cells within the TME. AMPK, an energy and nutrient sensor, is critical for survival of activated CD8^+^ T cells [35] and plays a key role in metabolic reprogramming of naïve to effector and effector to memory T cells [23, 36]. As shown in Figure 3D, AMPK was differentially regulated within IDO^−/−^ CD8^+^ T cells compared to WT. CD8^+^ T cells from IDO^−/−^ mice showed increased AMPK activation and amplified signals upstream and downstream of AMPK. Activation or phosphorylation of PFKFB2 can turn on glycolysis which is necessary for the conversion of glucose to lactate [7, 21]. Activated CD8^+^ T cells, like tumor cells, typically utilize glycolysis to generate energy [7]. CD8^+^ T cells from IDO-deficient mice, as well as combination therapy treated mice, produced significantly higher levels of IFN-γ and lactate, a metabolic end product of glycolysis [7], (Supplementary Figure S7B). Importantly, cellular lactate concentrations were reduced in tumor-purified LLCs from IDO^−/−^ mice (Supplementary Figure S7C). Notably, PFKFB2 activation was not different between WT and IDO^−/−^ LLCs (Supplementary Figure S6B). These data suggest that CD8^+^ T cells from tumor-bearing IDO^−/−^ mice are more likely to have enhanced anti-tumor effects compared to those from tumor-bearing WT controls. Taken together, AMPK-driven metabolic alterations in MDSCs and CD8^+^ T cells of IDO^−/−^ mice may cause perturbations in the TME that contribute to the reduced tumor burden, percentages of MDSCs, and percentages of exhausted CD8^+^ T cells in IDO^−/−^ mice.

Our studies provide the first evidence linking AMPK and IDO pathways in immune cells. Since combination therapy altered the metabolic status of memory T cells [15], we anticipated that this IDO-inhibiting therapy could also regulate AMPK and mTOR pathways in the TME.

### Combination therapy inhibits GCN2, mTOR, and AMPK pathway activation

**Figure 4:**
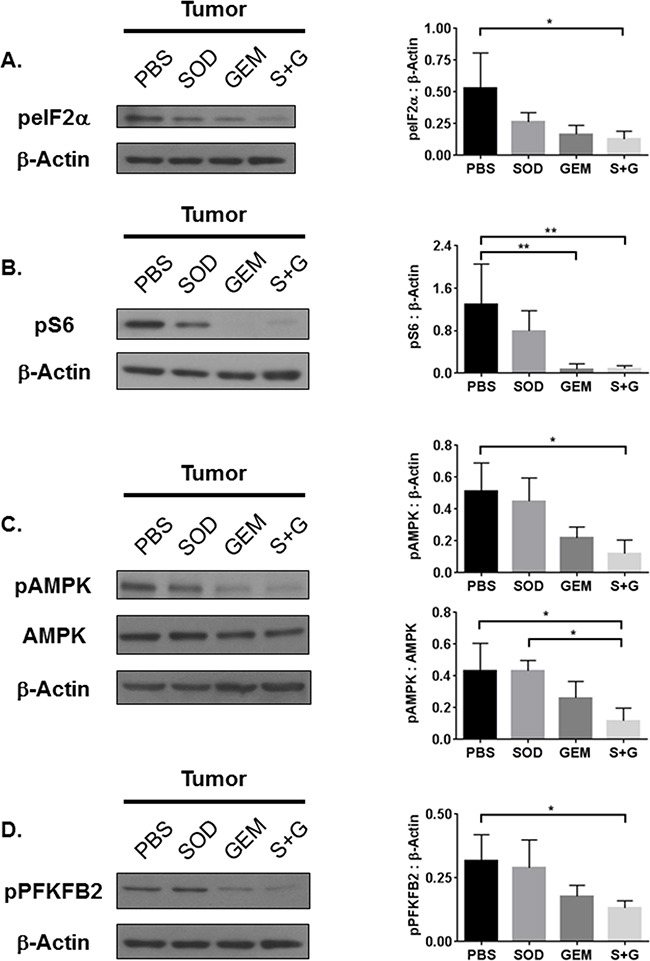
Combination treatment reduces tumor tissue GCN2, mTOR, and AMPK activation Western Blot analyses of whole tumor lysates reveal that **A.** eIF2α, **B.** S6, **C.** AMPK, and **D.** PFKFB2 activation are inhibited by combination treatment. Densitometry data, as a ratio of phosphorylated protein to β-Actin or total protein expression, are from 3-4 replicate experiments and assessed by one-way ANOVA with Tukey's post-test, *P<0.05, **P<0.01.

Next, we determined whether the combination therapy-mediated reduction of IDO expression and activity would modulate downstream amino acid-sensing pathways, including GCN2, mTOR, and AMPK. Downstream of GCN2, peIF2α was reduced in tumor tissues from combination therapy treated mice (Figure 4A). Even though combination therapy inhibits the IDO pathway and Trp deficiency signal peIF2α, it does not restore Trp sufficiency signaling via mTOR (Figure 4B). Consistent with our earlier findings in IDO^−/−^ mice, these data suggest that activation of the IDO pathway can be regulated independently of the Trp sufficiency signal pS6, as previously reported [10].

To rule out whether AMPK activation was responsible for the impaired activation of mTOR, we measured levels of phosphorylated and total AMPK. Phosphorylation of AMPK was reduced by combination treatment while the total AMPK levels remained unchanged (Figure 4C). As shown in Figure 4D, downstream phosphorylation of PFKFB2 was also inhibited. We concluded that combination therapy affects both Trp metabolism and cellular energy metabolism, which could be influenced by immune cells in the TME.

### Combination therapy inhibits IDO pathway activation in MDSCs, tumor cells, and CD8^+^ T cells

**Figure 5:**
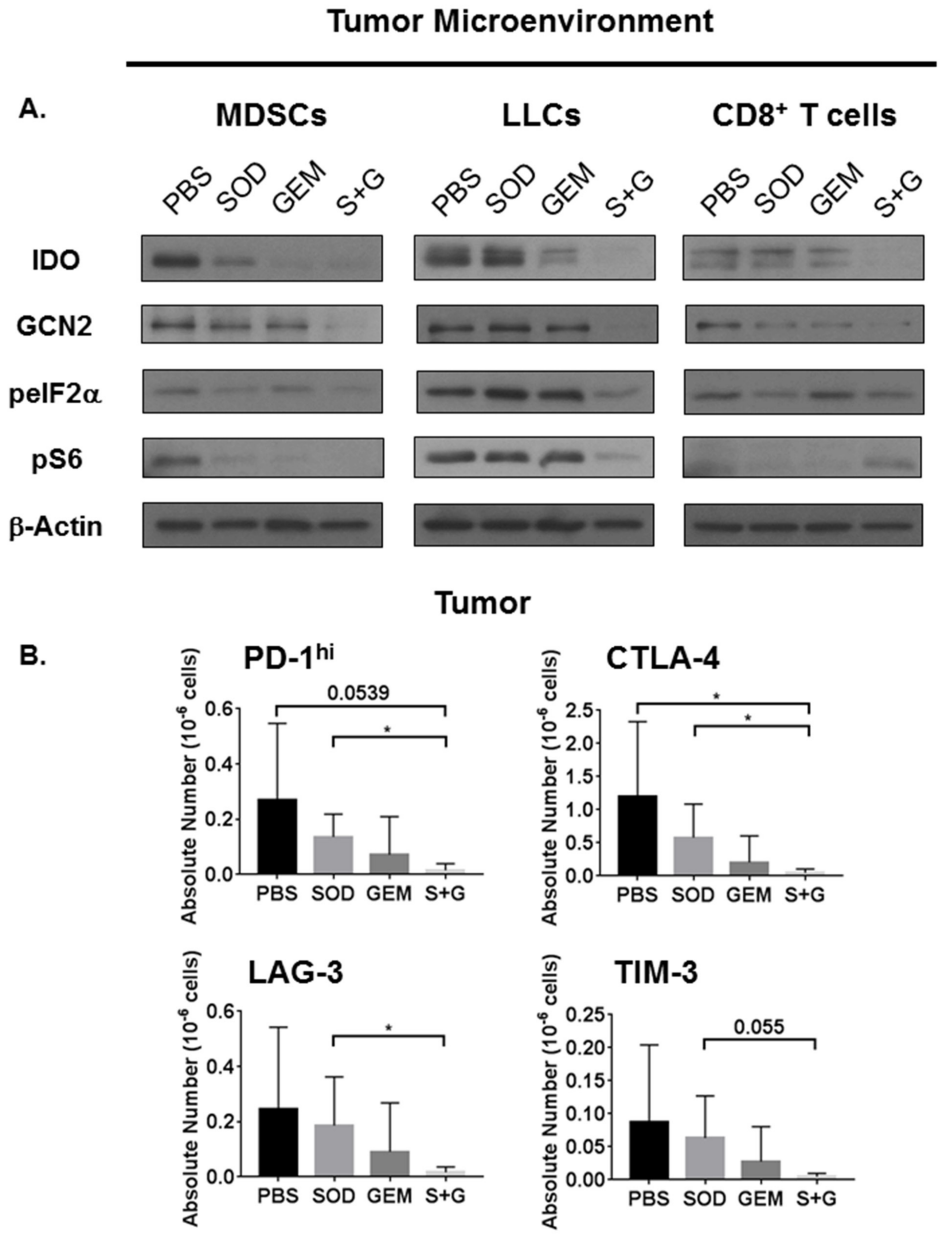
Combination treatment inhibits IDO and mTOR activation in MDSCs and LLCs but restores mTOR activation only in CD8^+^ T cells Combination therapy reduces expression of immune check point molecules. On day-14 post-*i.c.* LLC challenge, WT mice receiving individual and combination treatment were sacrificed and tumors were digested. Tumor-purified CD45^+^Gr1^+^CD11b^+^ MDSCs, CD45^+^CD8^+^ T cells, and CD45^−^ LLCs were isolated by FACS. CD8^+^ T cells were expanded and prepared as lysates. **A.** Combination therapy reduces the expression of IDO, GCN2, and phosphorylation of both eIF2α and S6 in MDSCs and LLCs. Combination therapy also impairs the IDO pathway in CD8^+^ T cells but restores S6 activation. **B.** On day-11 post *i.c.* LLC challenge, flow cytometric analyses of tumor CD8^+^ T cells also demonstrates that combination therapy treated mice have fewer absolute numbers of CD8^+^ T cells expressing checkpoint molecules PD-1^hi^, CTLA-4^+^, LAG-3^+^, and TIM-3^+^ (n=4-5 mice/group). Absolute numbers are compared by student's unpaired t-test with Welch's correction, *P<0.05.

To determine the signaling pathways altered in response to individual and combination therapies, we purified MDSCs, CD45^−^ LLCs, and CD8^+^ T cells from tumors and assessed activation status of IDO, GCN2, and mTOR pathways. We found that combination therapy inhibits IDO and GCN2 in all three cell types, but it reduces mTOR pathway activation only in MDSCs and LLCs (Figure 5A and Supplementary Figure S8). This broad inhibition of IDO by combination therapy led to the activation of Trp sufficiency signal pS6, specifically in CD8^+^ T cells and not in MDSCs or LLCs (Figure 5A and Supplementary Figure S8). Interestingly, since mTOR was activated in CD8^+^ T cells following combination therapy, robust activation of AMPK was not observed in CD8^+^ T cells (Supplementary Figure S7A).

These results support our initial observation that Trp sufficiency and deficiency signals must be mutually exclusive within these cell types. Phosphorylation of S6, by combination treatment in CD8^+^ T cells, can also positively regulate translation of IFN-γ even when glucose levels are low [37]. We found that IFN-γ secreted by CD8^+^ T cells from combination therapy treated mice is higher (Supplementary Figure S7B), consistent with elevated mTOR activation in these cells. Additionally, higher lactate levels in these cells persisted during T cell expansion (Supplementary Figure S7B), consistent with our previous observations [15].

### Combination therapy downregulates immune checkpoint molecules in the TME

To address whether alterations in checkpoint molecules in IDO-deficient mice would be recapitulated in response to combination therapy, we analyzed CD8^+^ lymphocytes within the tumor for immune checkpoint molecules PD-1, CTLA-4, LAG-3, and TIM-3. These checkpoint molecules were reduced by combination therapy in CD8^+^ T cells residing within the tumor (Figure 5B). This impairment of immune checkpoint molecules on CD8^+^ T cells may contribute to the enhanced activation and metabolism to promote anti-tumor responses.

### Depletion of MDSCs and CD8^+^ T cells does not promote tumor growth following combination therapy treatment

**Figure 6:**
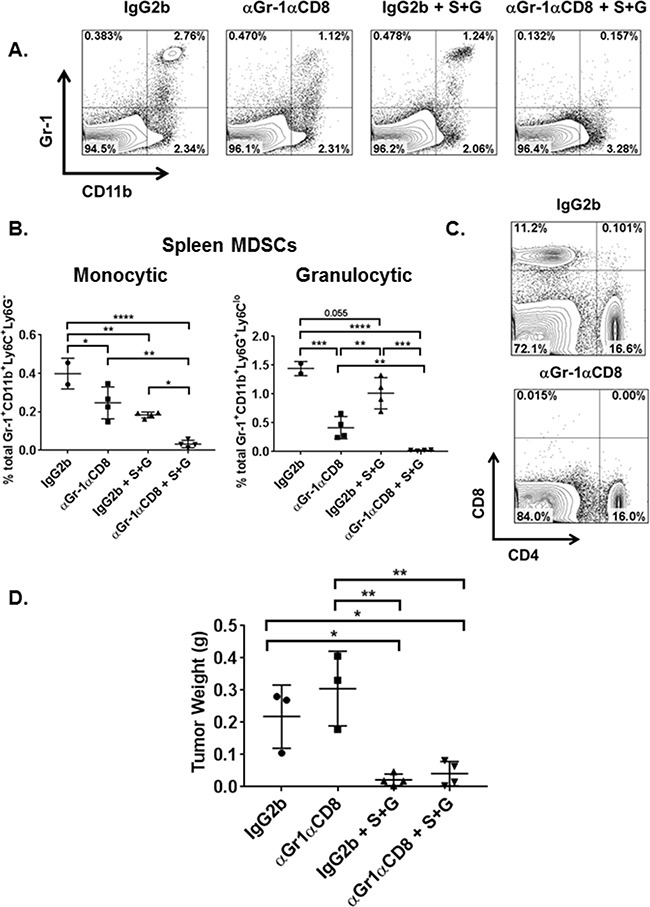
Both combination therapy and MDSC depletion reduce overall tumor burden and splenic MDSC subsets On day-3 following tumor implant, WT mice (n=3-4 mice/group) were administered depleting antibodies (BioXCell) for Gr-1 and CD8a or IgG2b isotype control by *i.p.* injection. On days 4 and 7, one group of Gr-1 and CD8a depleted mice (αGr-1αCD8) and IgG2b mice were also treated with combination therapy (S+G) by *i.p.* route. Mice were sacrificed on day-9 when spleens and tumor nodules were collected. Spleens were prepared for flow cytometric analyses of Gr-1^+^CD11b^+^ granulocytic and monocytic MDSCs as well as CD8^+^ and CD4^+^ T cells. **A.** FACS analyses of splenic tissue confirmed MDSC depletion in αGr-1αCD8, combination therapy alone (IgG2b + S+G), and αGr-1αCD8 + S+G mice compared to isotype control (IgG2b). **B.** Within the spleen, total percentages of monocytic and granulocytic MDSCs were reduced in IgG2b + S+G mice compared to isotype alone while αGr-1αCD8 + S+G mice demonstrated a significant impairment of spleen MDSC subsets compared to both αGr-1αCD8 and IgG2b + S+G mice. Monocytic MDSC data are compared by one-way ANOVA with Tukey's post-test, *P<0.05, **P<0.005, ****P<0.0001. Granulocytic MDSCs are compared by one-way ANOVA with Tukey's post-test for IgG2b vs αGr-1αCD8, αGr-1αCD8 vs IgG2b + S+G, and IgG2b + S+G vs αGr-1αCD8 + S+G, **P<0.01, ***P<0.0005, ****P<0.0001, and by student's unpaired t-test for IgG2b vs IgG2b + S+G and αGr-1αCD8 vs αGr-1αCD8 + S+G, **P<0.01. **C.** Characterization of CD8^+^ and CD4^+^ T lymphocytes in the spleen also confirmed the depletion of CD8^+^ T cells in αGr-1αCD8 mice. **D.** Compared to IgG2b isotype control and αGr-1αCD8 mice, the addition of S+G significantly reduced tumor burden, respectively. Tumor weights are compared by one-way ANOVA with Tukey's post-test, *P<0.05, **<0.005.

Gemcitabine has been reported to have cytotoxic effects on proliferating tumor cells, which can directly influence tumor cell growth as well as depleting effects on MDSCs. To identify whether the observed effects by combination therapy are restricted to metabolic signaling alterations with immune cells, we depleted both MDSCs and CD8^+^ T cells using depleting antibodies and measured tumor burden compared to isotype and combination treatment controls. We confirmed depletion of MDSCs in the spleens of treated mice by flow cytometric staining for Gr-1^+^CD11b^+^ cells (Figure 6A). While combination therapy significantly reduced monocytic and granulocytic MDSCs compared to isotype control, when MDSCs and CD8^+^ T cells were depleted and combination therapy was also administered, monocytic and granulocytic MDSCs were significantly reduced compared to combination therapy alone (Figure 6B). CD8^+^ T cell depletion was confirmed in the spleens of these mice (Figure 6C). Our data show that combination therapy significantly reduces tumor burden even when CD8^+^ T cells and MDSCs are depleted compared to mice which did not receive combination therapy. This reduction in tumor burden was significantly higher than mice depleted with both αGr-1 and CD8 ^+^ T cell depletion alone (Figure 6D). Immune cell depletion alone was not significantly different from isotype alone, suggesting that combination therapy must act directly on tumor cells within the TME. This is also evident in Figure 5A where combination therapy is the only therapy that is most effective at reducing IDO expression and signaling in LLCs purified from tumor. Taken together, combination therapy exerts effects not only on MDSCs and CD8^+^ T cells within the TME but also directly on the LLCs.

## DISCUSSION

Among the emerging hallmarks of cancer, tumor immune evasion represents a unique target for the development of therapies designed to restore anti-tumor T cell functions [38]. Although immune-modulatory therapies appear promising, the precise mechanisms and signaling pathways subverted in immune cells, such as activated T cells and MDSCs, remain unclear. Monoclonal antibodies directed against CTLA-4 and PD-1/PD-L1, in addition to CAR T cells, are not exempt from IDO-mediated immune resistance mechanisms [39–41]. Thus, novel therapies that target the IDO pathway, while enhancing T cell functionality and responsiveness, may be superior to existing immunotherapy strategies. This stresses the impending need to develop multi-modal therapies combining checkpoint inhibitors or T cell-modifying treatments. Elucidating the signaling pathways employed by immune cells within the TME may shed light on the manner in which host defenses and tumor cells respond to immune-modulating combination treatments. An understanding of how these pathways act in concert with each other can facilitate the development and design of improved drug-targeting strategies.

MDSCs contribute to immunosuppression in the TME and are associated with poor prognosis and tumor burden in cancer [30, 42]. MDSCs modulate arginine metabolism [42–44] and have recently been implicated in the regulation of Trp catabolism in the TME, generating Kyn which inhibits cytotoxic immune responses [4, 5, 34]. In our studies, MDSCs were the main contributors to IDO expression in the TME. Similarly, IDO expression in murine and human MDSCs has been attributed to tumor progression and immune escape [24–27, 32]. Our data demonstrate that IDO was expressed at similar levels in lung tumor nodules and purified tumor cells from both the WT and IDO^−/−^ tumor-bearing mice. Despite this, we found reduced tumor burden and MDSC infiltration in the IDO-deficient mice. This indicates that the decrease in tumor burden was attributable to IDO deficiency in TME-infiltrating cells rather than tumor cells themselves. Reduced MDSC infiltration in IDO^−/−^ mice suggests that IDO impairment within this non-tumor cell population impedes MDSC-driven tumor growth. This is consistent with the predominant IDO expression in tumor-associated MDSCs. These findings are substantiated by reduced GM-CSF in the tumor-bearing lungs of IDO-deficient mice, indicating that MDSC-promoting factors, including IDO and GM-CSF, support tumor growth. IDO deficiency in MDSCs abrogates these effects, replicated by the administration of combination treatment. The observation that IDO-deficient bone marrow-differentiated MDSCs are more apoptotic emphasizes the importance of IDO in sustaining MDSC proliferation. These data are consistent with another report that IDO deficiency mitigated lung tumor outgrowth, lesion formation, and MDSC suppressive function, improving overall survival in both KRAS-induced, spontaneous lung cancer and metastatic breast cancer models [6].

Previous studies investigating IDO signaling show that IDO regulates downstream GCN2 and mTOR pathways [10]. Some studies have examined the expression of IDO within immune cells following chemotherapy [26, 45, 46]. Our studies establish a relationship between IDO and nutrient-sensing mechanisms in the TME and further elucidate the alterations in cellular signaling within immune cells of the TME during IDO deficiency, by genetic and therapeutic strategies. Compared to WT controls, IDO-deficient MDSCs isolated from tumor-bearing mice showed downregulated GCN2 and AMPK signaling, independent of mTOR activation. Taken together with our observations of enhanced apoptosis in IDO-deficient MDSCs, AMPK downregulation in the absence of IDO may influence the metabolic plasticity and ability of MDSCs to sustain the hostile TME. We also address here the reciprocal relationship between IDO and AMPK activation in the TME. Since the MDSCs are the main IDO contributors in the TME, reduction of IDO in MDSCs may influence AMPK activation in CD8^+^ T cells. This inverse relationship was observed in IDO-deficient CD8^+^ T cells, with upregulation of GCN2 and AMPK activation, also independent of mTOR. Whether the absence of IDO in T cells mediates other compensatory downstream GCN2 activation pathways to alter metabolic signaling cues remains an area of further investigation. To overcome T cell inactivation induced by Trp depletion and Kyn accumulation, our studies show that effector T cells switch to alternate bioenergetics mechanisms to maintain survival in a nutrient-depleted TME. A regulatory role for AMPK is justified as it acts as a major regulatory kinase governing metabolic plasticity, survival, and memory formation of T cells during nutrient limitation [36, 37]. Enhanced activation of this pathway can improve the cytotoxicity and lifespan of these cells, potentially impeding continued tumor outgrowth. Additionally, the reduced infiltration of MDSCs resulting in reduced oxidative stress will decrease immunosuppression and promote expansion of effector CD8^+^ T cells shifting the balance in favor of anti-tumor immunity.

CD4^+^ and CD8^+^ T cells from spleens of IDO^−/−^ mice exhibit reduced PD-1^hi^ cell surface expression, corresponding to less T cell exhaustion. Tumor-derived IDO^−/−^ CD8^+^ T cells also demonstrate increased lactate and IFN-γ production. This reversal of T cell exhaustion may contribute to the tumor burden reduction in IDO^−/−^ mice. These findings are especially pertinent as IDO has been shown to be a resistance mechanism that can impede the efficacy of checkpoint blockade therapies [39]. We observed no discernable difference in bioenergetics signaling pathways within tumor cells, suggesting that altered metabolic pathways in IDO-deficient MDSCs and CD8^+^ T cells keep tumor progression and metabolism in check. These results represent a novel finding that, in the absence of IDO within immune cells in the TME, AMPK functions as a key regulator of cellular metabolism.

We have also investigated the tumor-immune cross talk in the TME following treatment with our IDO-inhibiting combination therapy. The direct targeting of IDO in both LLCs and MDSCs led to the activation of mTOR (Trp sufficiency) signaling in CD8^+^ T cells leading to enhanced IFN-γ and lactate production in T cells. Importantly, when Trp sufficiency signal was activated in CD8^+^ T cells, we did not see a significant activation of AMPK, suggesting that mTOR activation was sufficient to enhance the effector function of the CD8^+^ T cells. We confirmed that combination therapy can directly impair tumor burden in the absence of MDSCs and CD8^+^ T cells suggesting that combination therapy has direct tumor-killing effects which may potentiate the efficacy of this therapy.

The expression of IDO has been shown to interfere with the efficiency of immunotherapies, including anti-CTLA-4, anti-PD-1/PD-L1, and anti-GITR [39]. When anti-PD-1/PD-L1 therapy was administered to IDO^−/−^ mice harboring B16F10 melanoma, they survived significantly longer compared to WT [39]. In our studies, tumor-bearing IDO^−/−^ mice have fewer PD-1^hi^ T cells that may reduce PD-1/PD-L1 interaction, prompting a more robust immune response. This is particularly interesting as PD-L1 has been identified to play a pivotal role in licensing glycolytic metabolism of tumor cells via AKT/mTOR, which limits nutrients needed for T cell survival and function [47].

Checkpoint blockade agents have been ineffective as single agents in IDO-overexpressing cancer models because they require a functional immune response to be elicited from tumor-residing CD8^+^ T cells [39, 48]. Many cancer patients diagnosed with late stage cancers exhibit CD8^+^ T cell hypo-responsiveness, which is primarily driven by nutrient competition between tumor and immune cells, limiting response to these kinds of therapies [47]. A study using human cancer cell lines has also identified IDO as a resistance mechanism to chemotherapeutics, including gemcitabine, making IDO a viable target [49]. Our combination therapy is advantageous by targeting MDSCs expressing IDO and enhancing CD8^+^ T cell metabolism necessary to achieve anti-tumor immunity, thus potentially making it successful without the use of additional IDO inhibitors.

From a clinical standpoint, the complex composition of the TME should not be overlooked and must be leveraged to enhance the efficacy of novel compounds and multi-modal therapies. As treatment regimens are becoming more individualized in the clinic, procedures to measure whether these agents can be directly associated with downregulation of IDO in both tumor and immune cells are warranted. Additionally, SOD mimetics may be used as adjuvants to MDSC-depleting therapies in order to directly target IDO in these cells.

**Figure 7:**
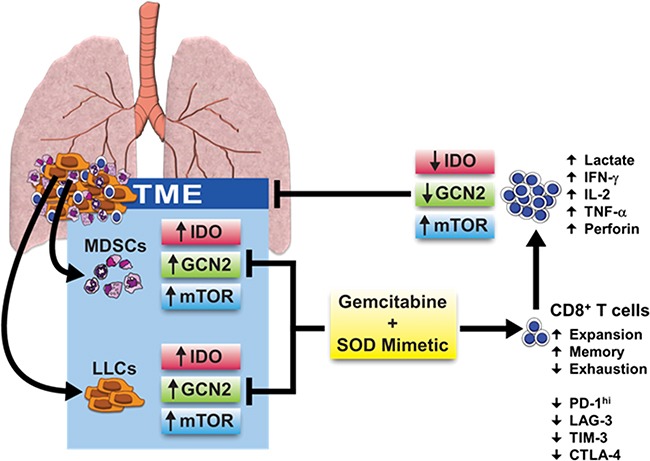
Effects of combination therapy on cellular signaling pathways and metabolic reprogramming in the TME A combination therapy of gemcitabine and a SOD mimetic inhibits IDO, GCN2, and mTOR pathway activation in IDO-expressing MDSCs and LLCs. This reverses immune suppression, reduces anti-tumor T cell exhaustion, and rescues mTOR activation in CD8^+^ T cells enabling their expansion and reprogramming towards a glycolytic state. Phosphorylation of S6 in CD8^+^ T cells licenses IFN-γ production necessary to elicit cytotoxic effects, diminishing tumor burden and restoring anti-tumor immunity.

Although we do not address the contribution of other cell types present in the heterogeneous TME, this study demonstrates the importance of cell-specific signaling that may influence cell-to-cell communication and direct metabolic instructions within the tumor. Based on these findings, we conclude that IDO, mTOR, and AMPK signaling pathways are differentially regulated in a cell-specific manner. In IDO-deficient mice, mTOR appears to be controlled independently of AMPK activation and dictates a new role for IDO in modulating this metabolic pathway. Importantly, combination therapy with gemcitabine and a SOD mimetic alters IDO, GCN2, and mTOR activation in MDSCs and LLCs in the TME, which can improve glycolytic metabolism and function of CD8^+^ T cells *in vivo* (Figure 7). Our depletion studies in mice also confirm an advantage of this combination therapy in directly diminishing tumor burden, even in the absence of both MDSCs and CD8^+^ T cells. These results demonstrate the novelty of this therapy, especially as IDO inhibitors and immune-modulating therapies are becoming more translationally relevant in the clinic [11, 50]. Overall, this study highlights the importance of metabolic cellular signaling and establishes a potential mechanism for a combination therapy of gemcitabine and a SOD mimetic that targets IDO in MDSCs and tumor cells to promote anti-tumor immunity against lung cancer.

## MATERIALS AND METHODS

### Cell culture

Mouse-specific Lewis lung carcinoma cells (LLCs) (ATCC) were propagated in Dulbecco's Modified Eagle Medium (DMEM) supplemented with 10% fetal bovine serum, 1 mM sodium pyruvate, 2 mM L-glutamine, 10 μg/ml penicillin-streptomycin and 0.1 mM non-essential amino acids (Life Technologies, Carlsbad, CA) as previously described [15]. LLCs were authenticated following ATCC guidelines based on morphology (rounded-loosely attached or floating), viability, recovery, and growth, and were confirmed one month before use in the experiments described. For *in vitro* experiments, LLCs were seeded in 6-well plates until 70-80% confluent then treated with fresh media containing 0-100 ng of recombinant mouse IFN-γ (eBioscience).

### Syngeneic mouse model of lung cancer

6-8 week old C57BL6 (Frederick Cancer Research and Development Center, MD) and B6.129-Ido1/J (Jackson Labs) (IDO^−/−^) mice were injected with LLCs via intracardiac (*i.c.*) or intravenous (*i.v.*) route into isoflurane (Fisher) anesthetized mice, as previously described [15]. C57BL6 mice were treated by intraperitoneal route (*i.p.*) with superoxide dismutase mimetic (SOD) (10 mg/kg) (Mn(III)tetrakis(1-methyl-4-pyridyl)porphyrin (MnTMPyP – Calbiochem), EMD Millipore, cat. no. 475872) [51] and/or gemcitabine (GEM) (60 mg/kg) (Sigma-Aldrich) at Days 3, 7, and 10 post-*i.c.* injection. For experiments involving the IDO inhibitor, 1-methyl-D-tryptophan (D1MT) (Sigma-Aldrich), 1.5 mg/150 ml/mouse solutions were prepared freshly by dissolving D1MT in 0.1 N NaOH then adjusting the pH to 7 with 0.1 N HCl. Injections were administered by *i.p.* route in the morning and again in the afternoon for a total of 3 mg/mouse/day of D1MT on days-3, 5, and 8 post-tumor implant. Mice were monitored and euthanized following UAB IACUC guidelines and protocols. Serum was collected post-exsanguination and this was followed by *i.c.* lung perfusion with 1X PBS prior to removing lung tissues. Nodules from the harvested tumor masses, lung surface, and chest cavity were dissected then individually counted. Tumor weights were determined by pooling all nodules prior to tissue digestion.

### Western blotting

Tissue homogenates from lung, tumor, spleen, and sorted cell lysates were prepared in either PBS or RIPA buffer containing protease inhibitors. Samples were normalized for total protein (Pierce BCA Protein Assay Kit, Thermo Scientific) and run on 4-15% Mini-Protean TGX (Bio-Rad) or on 10% SDS-PAGE gels. Gels were transferred to 0.45 μm Immobilon-P PVDF membranes (EMD Millipore), blocked in either 5% non-fat dry milk (Lab Scientific) or Bovine Serum Albumin (BSA) (Fisher Scientific), and probed with antibodies overnight at 4°C. Blots were incubated for 1 hr at room temperature in secondary anti-rabbit or anti-mouse HRP-conjugated antibodies (Promega) prior to imaging with Luminol Reagent (Santa Cruz Biotechnology, Inc. and Millipore) and Kodak X-ray film (Z&Z Medical, Inc.). Antibodies used in immunoblots were peIF2α Ser51, GCN2, pLKB1 Ser428 (C67A3), pAMPK Thr172 (40H9), AMPKα (total AMPK) (Cell Signaling), pS6 Ser240/Ser244, pPFKFB2 Ser466, IDO Clone 10.1 (EMD Millipore, LifeSpan BioSciences, Inc.), IDO2 (Rockland Immunochemicals, Inc.), TDO2 (clone E-14, Santa Cruz), and actin (Sigma-Aldrich) at manufacturers' recommended dilutions.

### Flow cytometry and cell sorting

As described previously [15], tumor tissues were minced on ice in serum-free Iscove's Dulbecco's Modified Eagle Medium (IDMEM) (Corning) and then digested in serum-free IDMEM supplemented with 2 mg/ml of collagenase B (Roche) and 0.02 mg/ml of deoxyribonuclease I from bovine pancreas (Sigma) at 37°C for 30 min. After digestion, suspensions were neutralized with sterile IDMEM containing 10% fetal bovine serum (FBS) (Peak Serum) filtered through 0.45 mm strainers. ACK lysis buffer (Quality Biological, Inc.) was added to cell pellets to lyse any red blood cells present and washed with PBS. Spleen cells were extracted by cutting the capsule in half and scratching out the pulp into a dish containing cold PBS. Tumor and spleen cell suspensions were filtered, treated with ACK, and incubated on ice for 30 min in 3% BSA in PBS containing purified rat anti-mouse CD16/CD32 (Clone 2.4G2) (BD Pharmingen) to block murine F_c_ receptors. Cells were then stained with Gr-1 PE/APC (Clone RB6-8C5), Ly6C PerCPCy5.5 (HK1.4), CD8a FITC/APC (53-6.7), CD45 PE (30-F11), CD4 PECy7 (GK1.5), CD279/PD-1 PerCP-eFluor710 (J43), CTLA-4 (UC10-4B9), LAG-3 (C9B7W), TIM-3 (8B.2C12), CD11b APCCy7 (M1/70), and Ly6G FITC (1A8) (BD Pharmingen) antibodies for an additional 30 min on ice protected from light. Cells were washed with PBS prior to flow cytometry on BD LSR-II or sorting on BD FACS Aria III cytometer (UAB Flow Cytometry Core Facility). Additionally, intracellular staining was performed on spleen and tumor CD8^+^ T cells for IL-2 (JES6-5H4), TNF-α (MP6-XT22), perforin (eBioOMAK-D) (eBioscience), and IFN-γ (XMG1.2) (BD Pharmingen). Cells were incubated in stimulating RPMI media containing Phorbol 12-myristate 13-acetate, or PMA, (0.1 mg/ml, Sigma), ionomycin (1 mg/ml, Sigma), and a 1:1000 dilution of Golgistop/Golgiplug (BD Biosciences). Following stimulation, cells were permeabilized using the BD cytofix/cytoperm kit (BD Biosciences) then stained for CD8 as well as intracellular cytokine markers. Data were analyzed using FlowJo software (version 7.6.5). For FACS purification, tumor cell suspensions were stained with antibodies recognizing CD45 (also known as leukocyte common antigen), Gr-1, CD11b, and CD8. Non-hematopoietic cells, representing LLC tumor cells, were derived from the CD45^−^ population. Within the same antibody cocktail, hematopoietic MDSCs were isolated from Gr-1^+^CD11b^+^ population within the CD45^+^ cell gate. Similarly, CD8^+^ T cells were purified from the CD8^+^ cells within the CD45^+^ cell gate.

### Differentiation of bone marrow MDSCs and apoptosis quantitation by flow cytometry

Femur and tibia bones were pooled from naïve WT and IDO^−/−^ mice and whole bone marrow suspensions were cultured in MDSC-differentiating medium, as previously described [52]. Cells were cultured in 6-well plates in complete RPMI media (described below) at a concentration of 5×10^6^ cells/ml, 1 mg/ml lipopolysaccharide (LPS) (from *Escherichia coli* 0111:B4, Sigma), and 10 ng/ml recombinant mouse granulocyte-macrophage colony-stimulating factor (GM-CSF) (eBioscience). After four days of incubation at 37°C, non-adherent cells were isolated and re-stimulated for another four days until samples were analyzed by flow cytometry. As described above, cells were first stained with Gr-1 APC and CD11b APCCy7 antibodies, washed in cold PBS, and then stained for Annexin V FITC and PI according to the manufacturer's protocol from the FITC Annexin V apoptosis detection kit I (BD Pharmingen).

### Ex vivo expansion of CD8^+^ T cells

Following tumor tissue digestion, CD45^+^CD8^+^ T cells were isolated by FACS. Cells were expanded *ex vivo*, as described before [15]. CD8^+^ T cells were cultured at 5×10^5^ cells per well in a U-bottom 96-well plate in RPMI 1640 media (Corning) containing 10% fetal bovine serum (FBS) (Peak Serum), 2 mM L-glutamine (Fisher), 1 mM sodium pyruvate (Life Technologies), 0.1 mM non-essential amino acids (Life Technologies), 10 mg/ml penicillin-streptomycin-amphotericin B (Fisher), 50 mM 2-mercaptoethanol (Sigma), 6.25 mg/ml recombinant mouse Interleukin-2 (IL-2) (eBioscience), 4 mg/ml purified hamster anti-mouse CD28 (clone 37.51) (BD Pharmingen), and 0.75 mg/ml purified anti-mouse CD3e (clone 145-2C11) (eBioscience). CD8^+^ T cells were expanded *ex vivo* with stimulating antibodies and IL-2 every four days for three-four passages until a visible cell pellet could be collected for lysate. Cell pellets were washed in 1X PBS prior to cell lysis in cold, 1X RIPA buffer followed by sonication on ice. Total protein was determined as described above.

### Lactate assay

Cell pellets were washed in PBS and stored at −80°C until use. Cell pellets were directly homogenized in lactate assay buffer and cleared by centrifugation. A lactate assay kit (BioVision, Inc.) was used to detect lactate concentrations within cell pellets or T cell supernatants.

### Enzyme linked immunosorbent assays (ELISAs)

Lung homogenates were prepared on ice in sterile PBS using a glass pestle tissue grinder and cleared by centrifugation. Cleared homogenates, as well as culture supernatants, were stored at −80°C until use. ELISA kits for mouse GM-CSF and IFN-γ were purchased from eBioscience.

### IDO enzymatic activity assay

An IDO enzymatic activity was performed using a protocol adapted from previously described methods [53, 54]. Murine samples used in the IDO activity assays were prepared in Dulbecco's Phosphate Buffered Saline (DPBS) (Fisher) containing protease inhibitors followed by sonication. 2X IDO reaction buffer (200 mg/ml catalase from bovine liver (Sigma), 800 mM L-tryptophan (Sigma), 100 mM DPBS, 40 mM L-ascorbic acid (Sigma), 20 mM methylene blue (Fisher), with and without tryptophan, was added to samples that were normalized for total protein by BCA assay. Samples were incubated at 37°C for 30 min and the reaction was stopped with 30% trichloroacetic acid (TCA) (Sigma). Samples were incubated again at 52°C for 30 min followed by centrifugation (5 min, 10,000 rpm, 4°C). Supernatants were used for spectrophotometric analyses by the addition of an equal amount of Ehrlich's Solution (Sigma). Samples were read at an absorbance of 480 nm and values were calculated based on a standard curve of L-Kynurenine (Sigma) from 0-30,000 mM. Final IDO activity values were determined by taking the difference between samples without tryptophan-containing IDO reaction buffer and those receiving tryptophan-containing IDO reaction buffer.

### Depletion experiments

LLC-implanted WT mice were injected *i.p.* on day-3 following tumor implant with 250 mg/100 ml/mouse of IgG2b isotype control (clone LTF-2), anti-Gr-1 (clone RB6-8C5), or anti-CD8a (clone 2.43) *InVivo*Plus antibodies purchased from BioXCell. Mice were then treated with individual or combination therapy, as described above, before sacrificing on day-9.

### Statistical analyses

Student's t-test (Unpaired, with or without Welch's correction), multiple t-tests (Holm-Sidak), one-way ANOVA, and two-way ANOVA were used for statistical comparisons. ANOVA post-hoc analyses were conducted by implementing Dunnett's and Tukey's multiple comparisons test along with adjusted P values. All graphs represent Mean ± SD. Comparisons between WT and IDO^−/−^ were evaluated by an unpaired student's t-test without Welch's correction unless otherwise stated. Results were calculated using GraphPad Prism 6.05 and Microsoft Excel 2010.

## SUPPLEMENTARY FIGURES


