# Supplementary material for: Indoleamine 2,3-dioxygenase regulates anti-tumor immunity in lung cancer by metabolic reprogramming of immune cells in the tumor microenvironment
Source: Oncotarget. 2016 Sep 26;7(46):75407–24. doi: 10.18632/oncotarget.12249 (PMC5340181; doi:10.18632/oncotarget.12249)
Supplement: Supplementary file 1 [file oncotarget-07-75407-s001.pdf]

Indoleamine 2,3-dioxygenase regulates anti-tumor immunity in lung cancer by metabolic reprogramming of immune cells in the tumor microenvironment

SUPPLEMENTARY FIGURES

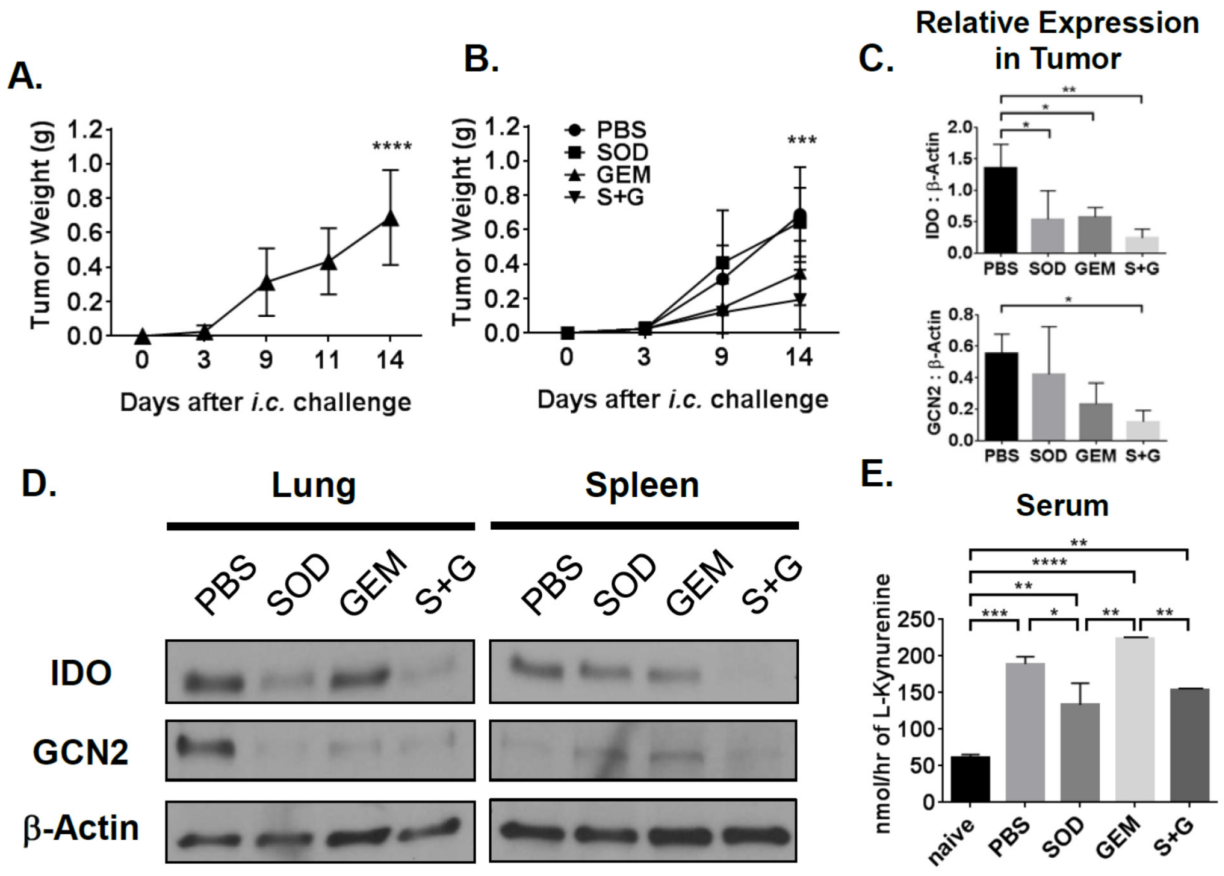

Supplementary Figure S1: Combination treatment diminishes tumor burden and inhibits IDO signaling and serum activity. C57BL6 mice were injected with LLCs via *i.c.* route. To assess tumor burden over time, mice were monitored daily and euthanized at days 0, 3, 9, 11, or 14. **A.** Tumor-bearing mice exhibit increasing tumor burden over time (n=3-14 mice/time point, replicate experiments, one-way ANOVA). Mice were treated with PBS, SOD mimetic (SOD), gemcitabine (GEM), or a combination of SOD and GEM (S+G). **B.** Tumor burden is reduced following combination therapy (n=3-11 mice/group/time point). Data are compared by two-way ANOVA with Tukey's post-test, ***P<0.0005, ****P<0.0001. **C.** Densitometry analyses of immunoblots for IDO1 and GCN2 expression in tumor tissue show reduction by combination treatment. Data are compared by two-way ANOVA with Tukey's post-test, *P<0.05, **P<0.01. **D.** Immunoblot IDO expression is reduced by combination therapy in lung and spleen tissues. **E.** Using serum samples collected at day-9, S+G and SOD alone lowered serum IDO activity levels. Values are calculated, as described, from duplicate wells. Data are compared by one-way ANOVA with Tukey's post-test, *P<0.05, **P<0.01, ***P<0.0005, ****P<0.0001.

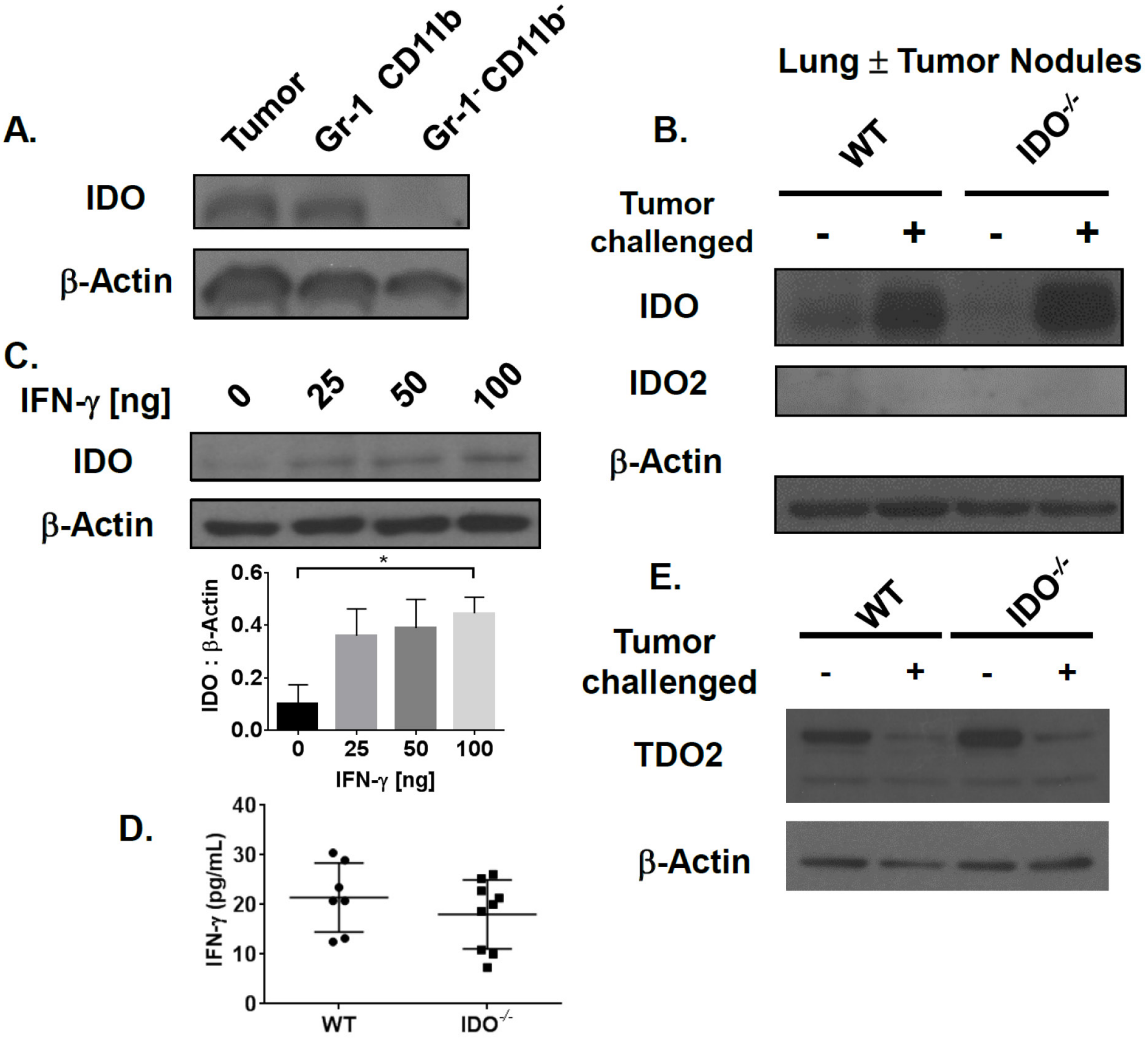

Supplementary Figure S2: IDO is expressed in tumor-recruited MDSCs. IDO is present in tumor-challenged WT and IDO-/- mice. IDO is expressed in LLCs following treatment with IFN-γ. TDO2 expression is reduced in tumor bearing mice. Following *i.c.* injection of LLCs at day-14, whole tumor tissue, and FACS-purified $Gr1^+CD11b^+$ and $Gr1^-CD11b^-$ cells were prepared for immunoblot analyses. **A.** Tumor tissue and $Gr1^+CD11b^+$ MDSCs express IDO while $Gr1^-CD11b^-$ cells do not. Lungs from naïve WT and $IDO^{-/-}$ mice were perfused with PBS and tissue lysates were prepared for immunoblot analyses. Similarly, lungs containing tumor nodules from WT and $IDO^{-/-}$ mice were collected 14 days post-LLC transplant. **B.** Only naïve WT lung tissue expresses IDO at very low levels while WT and $IDO^{-/-}$ mice upregulate IDO expression, and not IDO2, after tumor implant. LLCs were cultured and treated with increasing concentrations of IFN-γ for 24 hours. Cells were washed with PBS then lysed for Western Blotting analyses. **C.** LLCs express low basal levels of IDO *in vitro* which is significantly induced by IFN-γ treatment. Data represent duplicate experimental replicates and are compared by one-way ANOVA with Dunnett's post-test, 0 ng vs 100 ng, $*P<0.05$. Lung homogenates were collected and prepared from tumor-bearing WT and $IDO^{-/-}$ mice at day-11 post-tumor implant. **D.** Using an ELISA, IFN-γ concentrations in lung homogenates do not differ between WT (n=7) and $IDO^{-/-}$ mice (n=9) with established lung tumors. Data are compared by student's unpaired t-test, P=0.17. **E.** Western Blot analyses of lung tissue from naïve and tumor implanted WT and $IDO^{-/-}$ mice probed with α-TDO2 antibody.

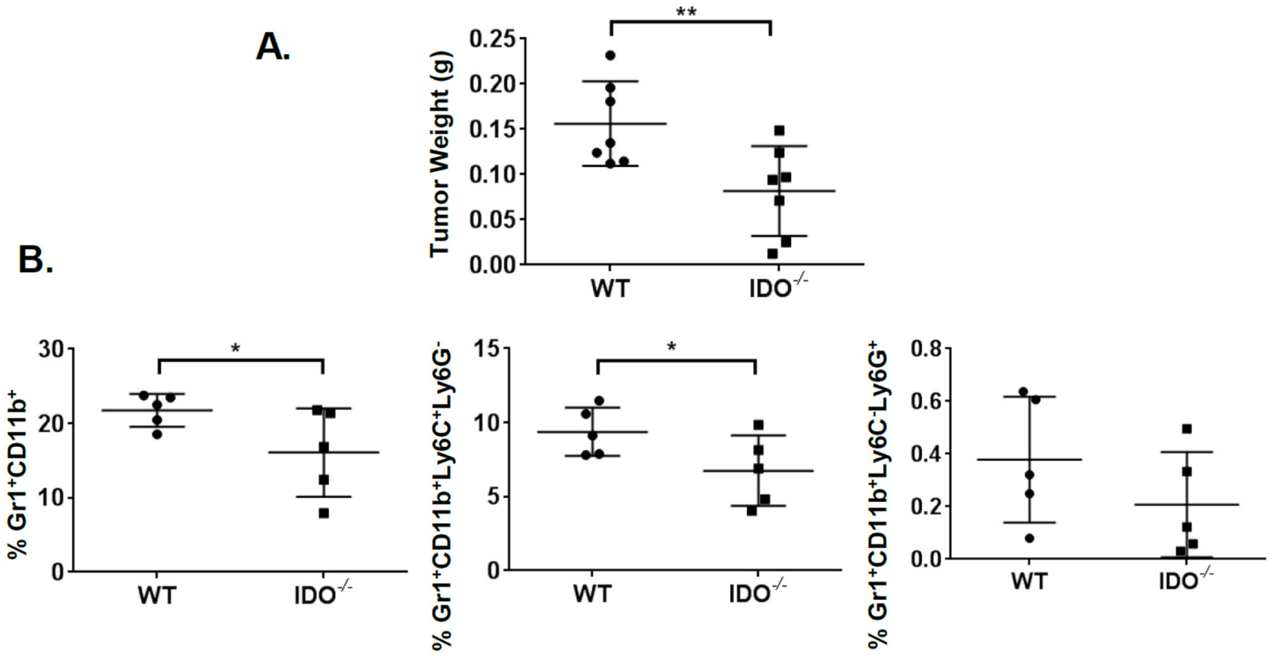

Supplementary Figure S3: IDO deficiency diminishes tumor burden along with tumor infiltration of MDSCs. On day-16 post-tumor injection via *i.v.* route, tumor nodules from each mouse were isolated and weighed. **A.** Tumor weights are significantly lower in IDO$^{-/-}$ mice compared to WT (n=7 mice/group, two independent experiments pooled). Tumor nodules were then digested into single cell suspensions for flow cytometry. **B.** From the live cell gate, total percentages of MDSCs, including monocytic and granulocytic MDSCs, are reduced in IDO$^{-/-}$ mice (n=5 mice/group). Data in A and B are compared by student's unpaired t-test, *P<0.05, **P<0.01.

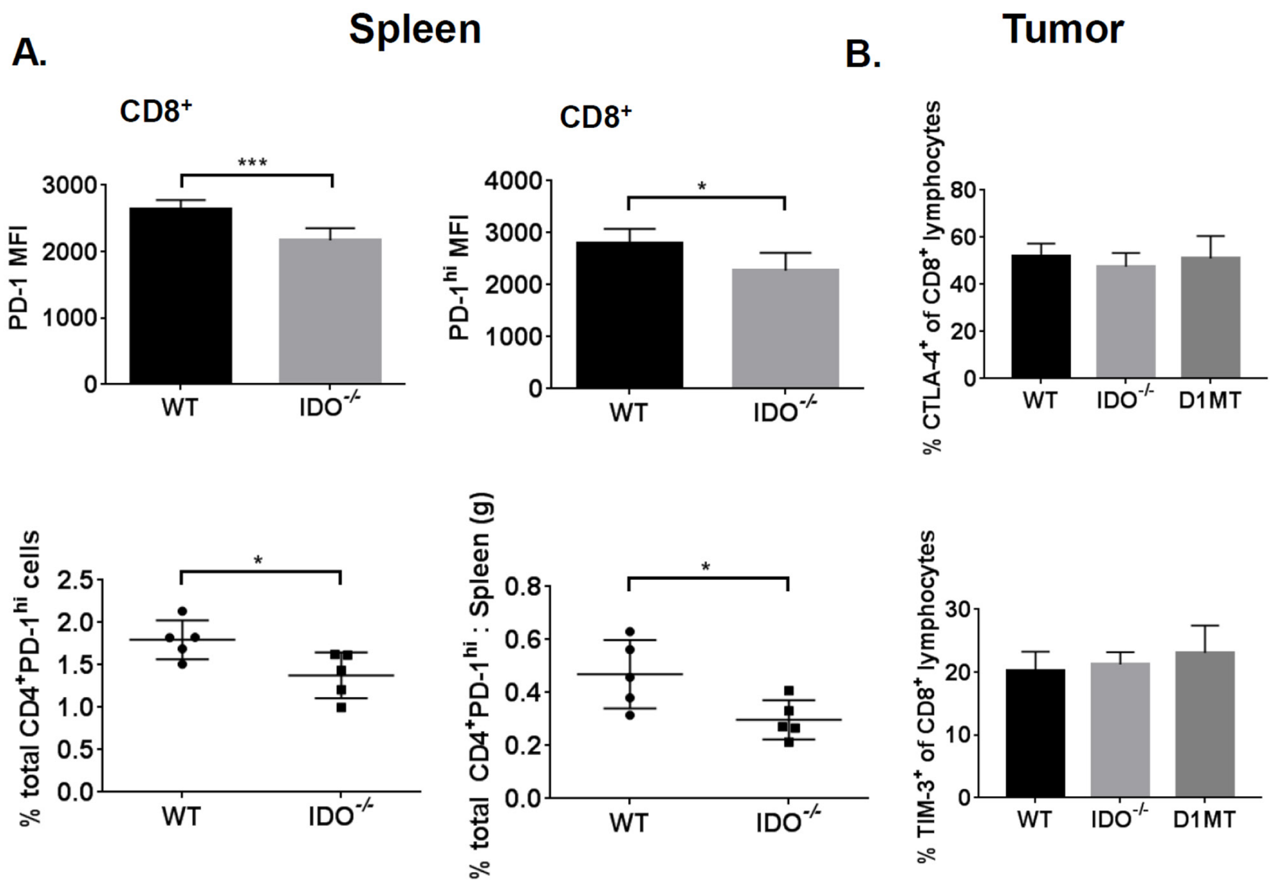

Supplementary Figure S4: IDO deficiency reduces expression and frequency of PD-1 on splenic T lymphocytes. On day-9 following i.c. tumor challenge, spleens were digested into single cell suspensions for flow cytometry. **A.** From the live cell gate, Mean Fluorescence Intensity (MFI) of $CD8^+PD\text{-}1^+$ and $CD8^+PD\text{-}1^{hi}$ lymphocytes, in addition to total percentages of $CD4^+PD\text{-}1^{hi}$ lymphocytes (and ratios to corresponding spleen weights), were significantly reduced in $IDO^{-/-}$ mice (n=5 mice/group). **B.** Within tumor tissue, there was no significant difference in $CD8^+$ checkpoint molecules, CTLA-4 or TIM-3, between WT, $IDO^{-/-}$, and D1MT treated mice (n= 4-5 mice/group). Data in A and B are compared using student's unpaired t-test, $^*P<0.05$, $^{***}P<0.001$.

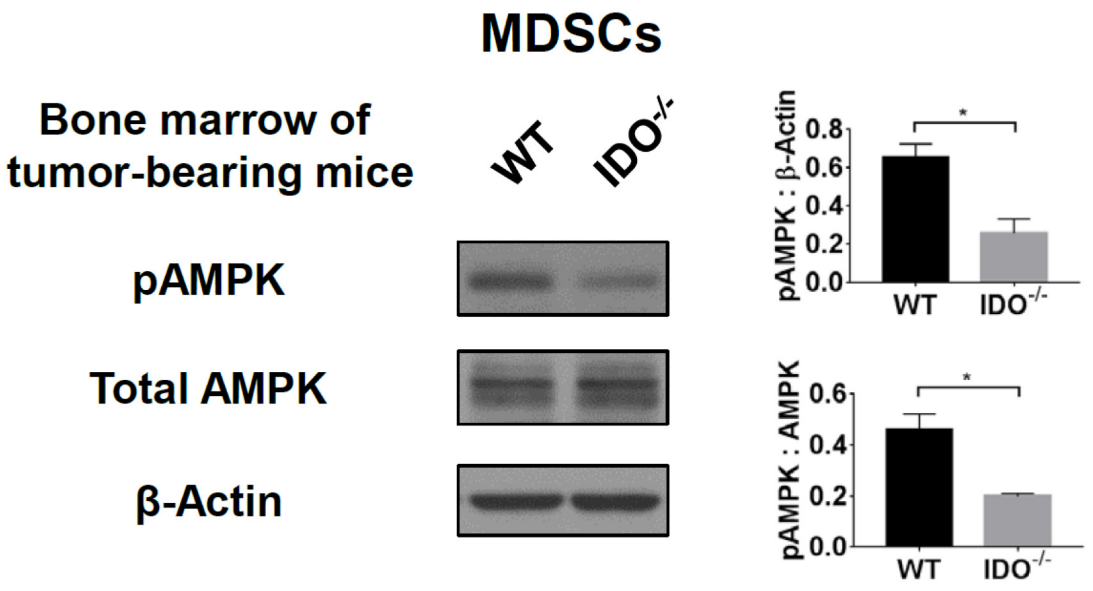

Supplementary Figure S5: Bone marrow MDSCs from IDO$^{-/-}$ mice have reduced AMPK activation. On day-14 post-tumor injection via *i.c.* route. MDSCs isolated from the bone marrow of IDO$^{-/-}$ mice exhibit reduced activation of AMPK compared to WT. Densitometry analyses for AMPK shows reduced activity in IDO-deficient MDSCs. Data are compared by student's unpaired t-test, *P<0.05.

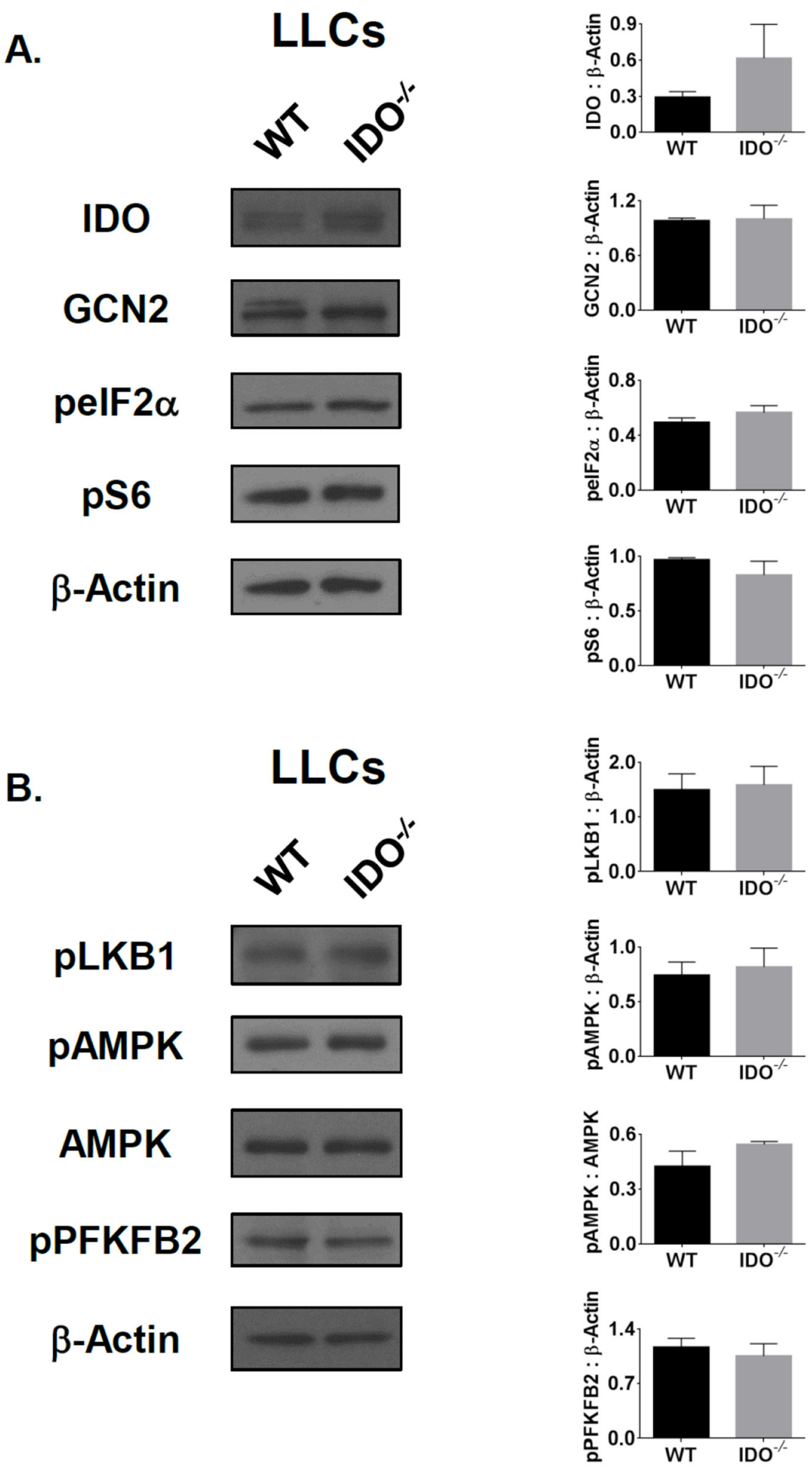

Supplementary Figure S6: Representative blots of IDO and AMPK pathway signaling within tumor-derived LLCs from WT and IDO$^{-/-}$ mice. CD45^{-} LLCs were immunosorted from digested tumor tissue from WT and IDO$^{-/-}$ mice at day-11 post-*i.c.* tumor implant. Cell lysates were normalized for total protein and analyzed by Western Blotting. LLCs show no difference in **A.** IDO or **B.** AMPK pathway proteins. Graph bars denote corresponding densitometry analyses of at least two experimental replicates. Data are compared using student's unpaired t-tests.

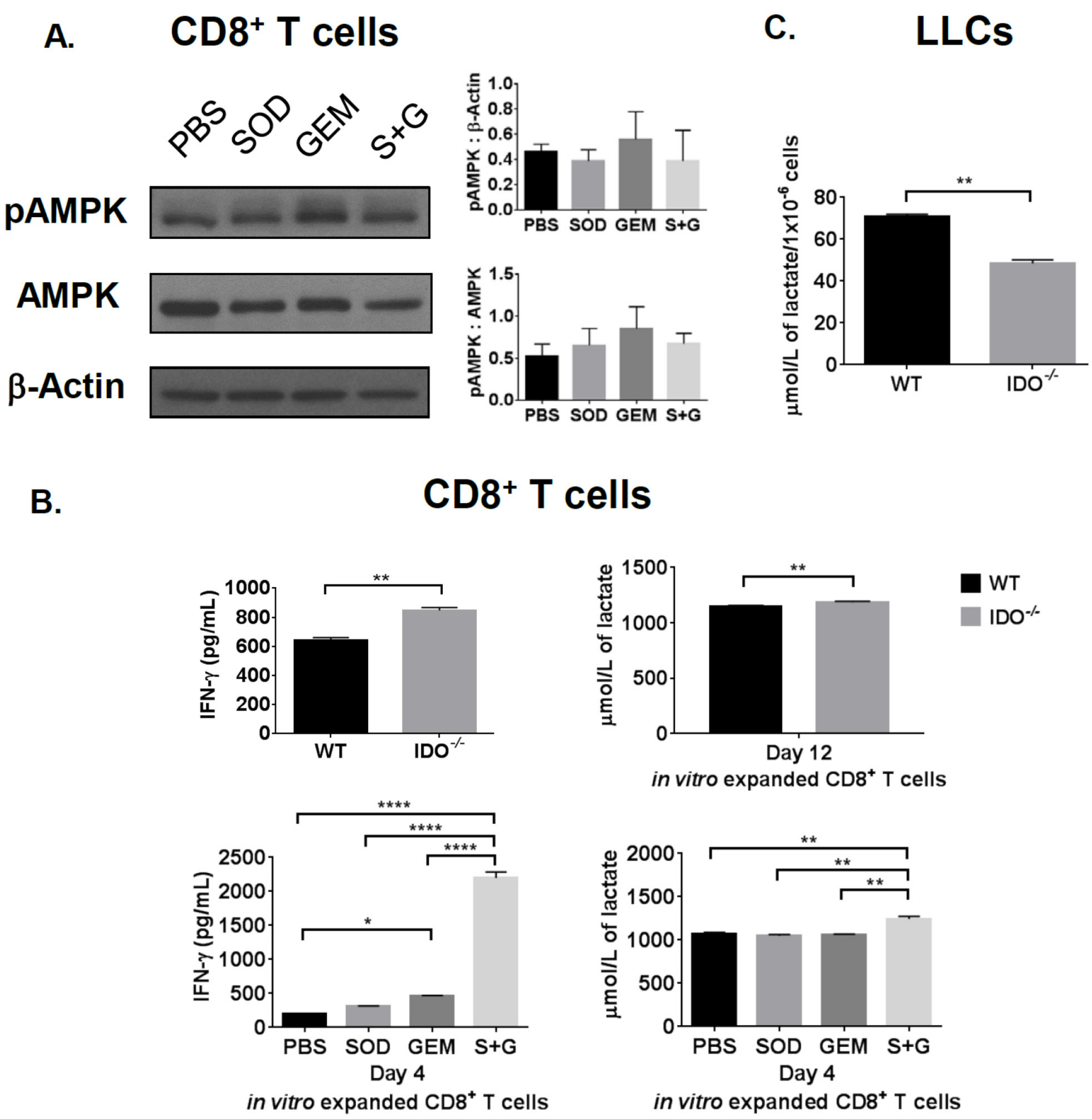

Supplementary Figure S7: IFN-γ and lactate production are enhanced with IDO deficiency or IDO impairment by combination therapy in T cells. IDO deficiency inhibits lactate production within LLC tumor cells. Tumor-purified $CD45^+Gr1^+CD11b^+$ MDSCs, $CD45^+CD8^+$ T cells, and $CD45^-$ LLCs were isolated by FACS. $CD8^+$ T cells were expanded and prepared as lysates. **A.** $CD8^+$ T cells from combination therapy treated mice have no difference in AMPK activation. Densitometry analyses were compared by one-way ANOVA with Tukey's post-test. **B.** $CD8^+$ T cells expanded from mice lacking IDO demonstrate enhanced IFN-γ (ELISA) and lactate production. Likewise, combination therapy increased supernatant IFN-γ (ELISA) and lactate production from $CD8^+$ T cells following expansion. WT vs $IDO^{-/-}$ samples were plated in duplicates or triplicates and data are compared using student's unpaired t-test, $^{**}P<0.01$. Treatment groups plated in duplicates are compared using one-way ANOVA with Tukey's post-test, $^*P<0.05$, $^{**}P<0.005$, $^{****}P<0.0001$. **C.** $CD45^-$ LLCs purified from tumors produce less lactate when isolated from $IDO^{-/-}$ mice. Data are plated in duplicates and are compared using student's unpaired t-test, $^{**}P<0.005$.

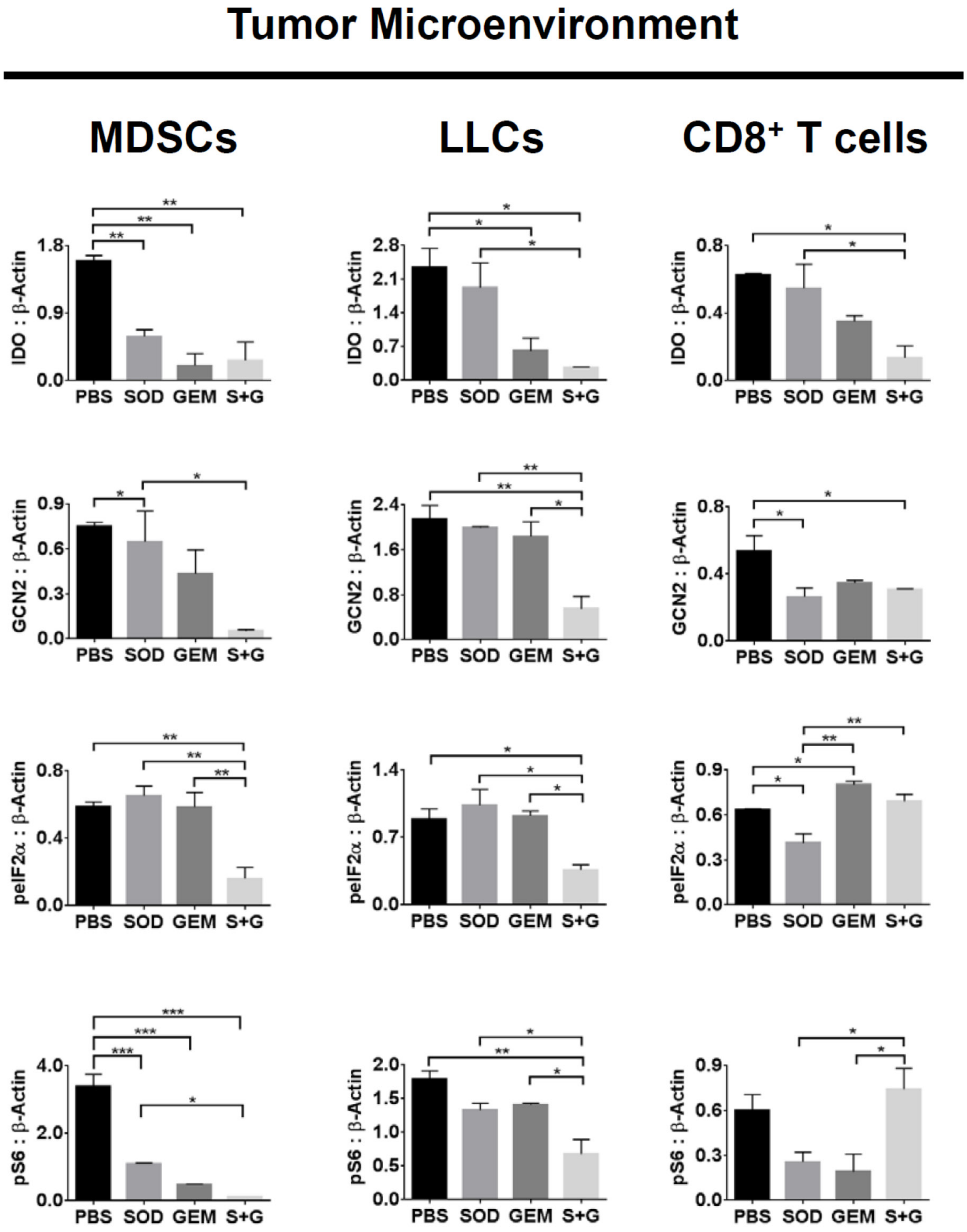

Supplementary Figure S8: Densitometry analyses from Western Blots comparing relative protein levels in tumor-derived MDSCs, LLCs, and CD8+ T cells from individual and combination therapy treated mice. Graph bars denote corresponding densitometry analyses of at least two experimental replicates. Values are compared using one-way ANOVA with Tukey's post-test, $^*P<0.05$, $^{**}P<0.01$, $^{***}P<0.001$.
